# Chondroitin sulfate proteoglycan serglycin influences protein cargo loading and functions of tumor-derived exosomes

**DOI:** 10.18632/oncotarget.20564

**Published:** 2017-08-27

**Authors:** Anurag Purushothaman, Shyam K. Bandari, Darshan S. Chandrashekar, Richard J. Jones, Hans C. Lee, Donna M. Weber, Robert Z. Orlowski

**Affiliations:** ^1^ Department of Lymphoma and Myeloma, The University of Texas MD Anderson Cancer Center, Houston, Texas, USA; ^2^ Department of Pathology, University of Alabama at Birmingham, Birmingham, Alabama, USA; ^3^ Department of Experimental Therapeutics, The University of Texas MD Anderson Cancer Center, Houston, Texas, USA

**Keywords:** serglycin, exosomes, multiple myeloma, chondroitin sulfate, protein cargo

## Abstract

Tumor cells produce and utilize exosomes to promote tumor growth and metastasis. Tumor-cell-derived exosomes deliver cargos that partially mimic the contents of the parent cell to nearby or distant normal or abnormal cells, thereby reprogramming the recipient cells to support tumor progression. Mechanisms by which tumor-derived exosomes subserve the tumor are under intense investigation. Here we demonstrate a critical role of the chondroitin sulfate proteoglycan serglycin in regulating the protein cargo and functions of myeloma cell-derived exosomes. Previous studies have shown that serglycin, the only known intracellular proteoglycan, functions mainly in the storage of basically charged components within the intracellular granules/vesicles *via* serglycin’s densely clustered, negatively charged glycosaminoglycan chains. Here we demonstrate that serglycin plays a critical role in the protein cargo loading of tumor-derived exosomes. Serglycin was detected in exosomes derived from cell culture supernatants of human myeloma cell lines and serum of myeloma patients. Mass spectrometry analysis of exosomal proteins identified significantly fewer protein components within exosomes derived from serglycin-knockdown myeloma cells than within exosomes from control cells. On gene ontology analysis, exosomes derived from serglycin-knockdown cells, but not from control cells, lacked many proteins that are required for mediating different cellular processes. In functional assays, exosomes from serglycin-knockdown cells failed to induce an invasive phenotype in myeloma cells and failed to promote migration of macrophages. These findings reveal that serglycin plays an important role in maintaining the protein cargo in tumor-derived exosomes and suggest that targeting serglycin may temper the influence of these exosomes on cancer progression.

## INTRODUCTION

One factor driving multiple myeloma is the interactions between myeloma cells and the bone marrow microenvironment. Recent progress in the management of multiple myeloma, a malignancy of plasma cells, has led to the realization that future therapies should aim to disrupt these interactions to improve patient outcomes [1, 2]. Exosomes, secreted membrane vesicles 30-120 nm in diameter, have emerged as important mediators of intercellular communications. Several studies have shown that exosomes secreted by tumor cells can affect survival, apoptosis, invasion, angiogenesis, resistance to therapy, and pre-metastatic niche formation [3-8]. Exosomes influence these processes by mediating signaling at the cell surface and by facilitating intercellular transfer of tumor-derived proteins and nucleic acids. We and others recently identified critical roles of myeloma-derived exosomes in promoting intercellular communications leading to enhanced osteoclast formation, angiogenesis, immunosuppression, and myeloma progression [9-13]. In addition a recent study demonstrated that exosomes from myeloma patients sera prime the bone marrow microenvironment to support survival and growth of primary multiple myeloma cells [14].

Glycosaminoglycans (GAGs) attached to proteoglycans (PGs) play an important role in the biology of exosomes. For example, heparan sulfate GAGs bind exosomes and function as cell surface receptors to mediate exosome internalization [15]. Furthermore, the heparan sulfate PGs of the syndecan family, through formation of a molecular complex with syntenin and ALIX (ALG-2-interacting protein X), help in exosome biogenesis [16]. We and others found that heparanase activates the syndecan-syntenin-ALIX pathway and promotes exosome secretion [12, 17]. Moreover, we recently showed that fibronectin present on the surface of exosomes acts as the ligand for cell surface heparan sulfate chains to bind exosomes [9]. In addition to heparan sulfate GAGs, yet another type of GAG that is ubiquitously expressed by various tissues and cells are the chondroitin sulfate (CS) GAGs.

We previously reported that the CS GAGs attached to the core protein of serglycin constitute the major component of the myeloma glycocalyx [18]. Though serglycin has long been considered as an intracellular PG located primarily in storage granules and secretory vesicles [19, 20], we and others found that myeloma cells constitutively secrete serglycin and that serglycin is the major PG released ( > 70% of the total) and the only CSPG expressed by myeloma cells [18, 21]. However, a mechanistic understanding of the extracellular role of serglycin in myeloma pathobiology remains incomplete. Studies in serglycin-knockout animals have shown that the sulfated and thereby negatively charged GAG chains of serglycin mediate storage of basically charged components (e.g., proteases, growth factors, and chemokines) within the intracellular granules/vesicles [19, 22-25]. Strong support to this notion came when two groups simultaneously showed that interference with GAG-modifying sulfotransferase NDST-2 (N-Deacetylase/N-Sulfotransferase 2), dramatically reduced storage of proteases in secretory granules of mast cells [26, 27]. The defective storage of proteases in the secretory granules of mast cells from serglycin-knockout mice is due not to mis-sorting into the constitutive pathway of secretion but rather to the defect in their retention within these granules. Therefore serglycin is critical for the maturation of secretory vesicles with an electron-dense core formation [20]. Densely clustered GAGs within the extensive stretch of serine-glycine repeats in the central part of the serglycin core protein enable this molecule to tightly pack large amounts of GAG-binding compounds within a small volume [28]. Interestingly, Braga *et al* observed that secretory granules of bone marrow cells from serglycin knockout animals lacked exosomes filled with electron-dense materials, compared to exosomes from their normal littermates [22]. This observation for the first time reveals the physiological relevance of a molecule in determining the protein repertoire within exosomes.

In the study reported here, we discovered that serglycin is present in exosomes derived from the cell culture supernatants of human myeloma cell lines and from the serum of myeloma patients. More importantly, consistent with the findings from serglycin-knockout animals, we discovered that exosomes from myeloma cells with serglycin knockdown had significantly fewer proteins than exosomes from serglycin-expressing control cells. Additionally, compared to serglycin containing exosomes, serglycin-null exosomes were less effective in altering tumor and host cell behavior. Our findings provide the first evidence of a critical role of serglycin in regulating the cargo and functions of tumor-derived exosomes and have a broad significance since a role for serglycin in different cancer progression (such as breast, lung, nasopharyngeal) is recently becoming apparent.

## RESULTS

### High serglycin expression in myeloma patients correlates with low survival rate

**Figure 1:**
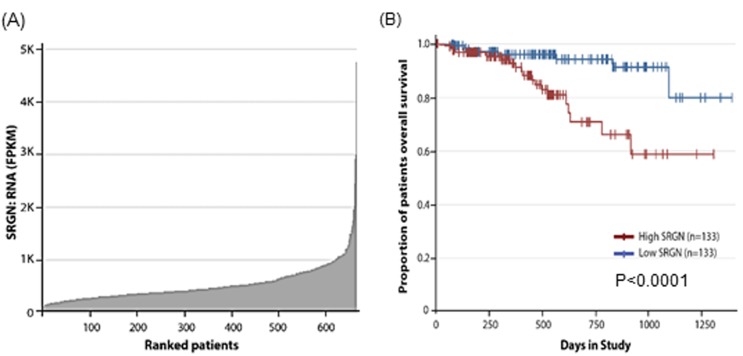
High serglycin expression in myeloma cells correlates with low survival rate **A.** Serglycin (SRGN) expression in tumor samples of myeloma patients (*n* = 664) obtained from the Multiple Myeloma Research Foundation Researcher Gateway (http://research.themmrf.org). **B.** Analysis of MMRF CoMMpass data showed significant correlation of serglycin expression with clinical overall survival. The plot shows the Kaplan-Meier survival probability over time between the bottom 20% of patients (with low expression of serglycin, *n* = 133) and the top 20% of patients (with high expression of serglycin, *n* = 133) of the 664 total patients in the dataset, *P* < 0.0001. Patients belonging to the high serglycin expression cohort had a worse survival compared to the low serglycin expression cohort.

To determine the extent of serglycin expression in myeloma patients, we analyzed the GEP data bases from the CoMMpass (Relating Clinical Outcomes in Multiple Myeloma to Personal Assessment of Genetic Profile) database interim analysis IA9 (http://research.themmrf.org), with the objective of assessing the effects of serglycin gene expression in 664 myeloma patients who have data available [29]. As shown in Figure 1A, we found a range of serglycin expression in these patients. Further, to evaluate the potential correlation between the serglycin expression and patient survival, we sorted the 664 patients by serglycin expression from low to high and performed Kaplan-Meier survival analysis to compare the bottom 20% of patients (with low expression of serglycin) and the top 20% of patients (with high expression of serglycin). A significant difference in survival between patients with high and low expression of serglycin were noted, with high expression of serglycin showing poorer prognosis (Figure 1B).

### Serglycin is present in multiple myeloma-derived exosomes

**Figure 2:**
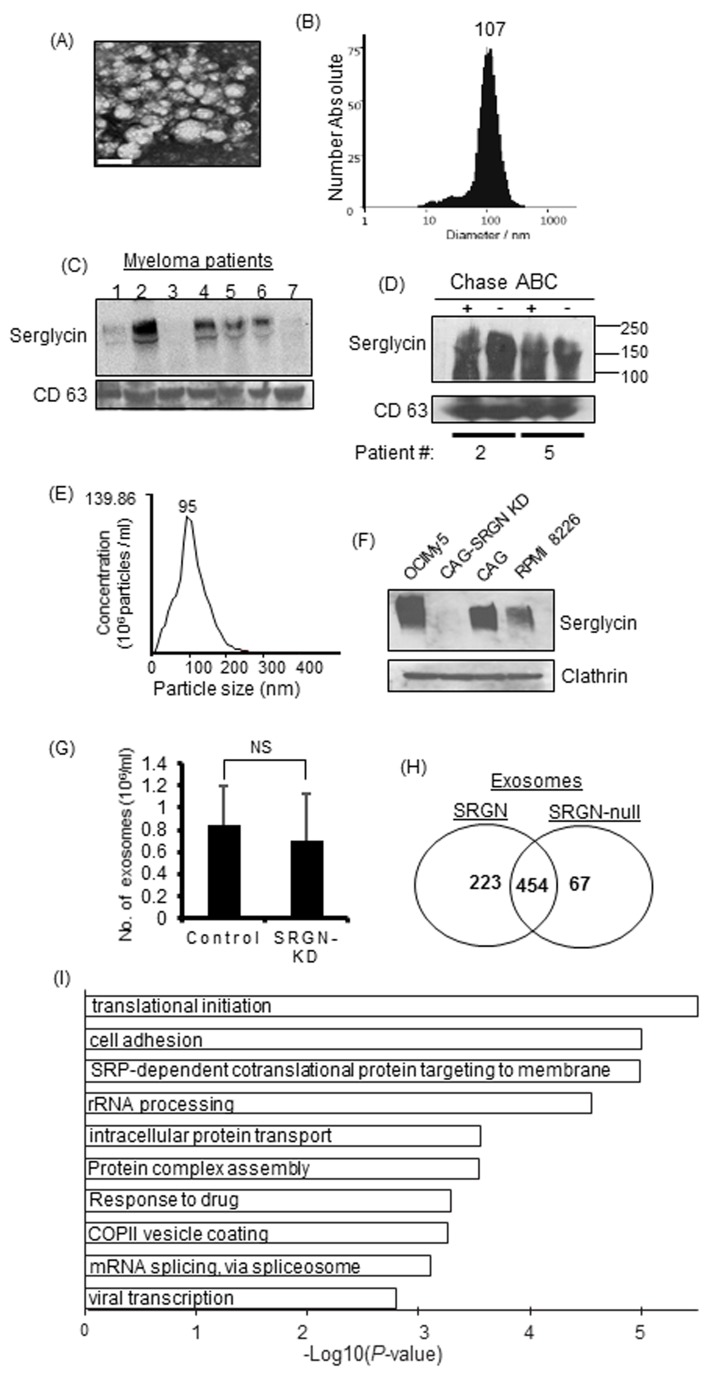
Serglycin is present in myeloma-derived exosomes and is required to maintain the protein cargo in exosomes **A.** Representative micrograph of exosomes from serum of patient with multiple myeloma. Exosomes were isolated from myeloma patient sera by ExoQuick precipitation followed by purification using microsphere beads. Electron microscopy showed that negative stained particles were of a size consistent with the size of exosomes. Bar = 100 nm. **B.** Quantification of the size and number of exosomes from a myeloma patient’s serum using a ZetaView exosome tracking analyzer. **C.** Western blot analysis of serglycin on exosomes isolated from the serum of 7 relapsed myeloma patients. **D.** Serum exosomes from two myeloma patients were treated with or without chondroitinase ABC (Chase ABC) and analyzed for serglycin and CD63 by western blotting. **E.** Quantification of the size and number of exosomes isolated from myeloma cell culture supernatants by nanoparticle tracking using a NanoSight 300. **F.** Exosomes purified from the 48-h conditioned cell culture supernatants of human myeloma cells OCIMy5, CAG (control shRNA), CAG with serglycin knockdown (CAG-SRGN-KD), and RPMI 8226 were analyzed for the presence of serglycin by western blotting. Clathrin, a marker for cell-derived vesicles, served as the loading control. **G.** Exosomes were isolated from 48-h conditioned media from control or SRGN-KD CAG cells and analyzed by nanoparticle tracking using a NanoSight 300. “NS” for non-significant. **H.** Venn diagram of proteins detected in SRGN and SRGN-null exosomes. SRGN-null exosomes had a lower diversity of proteins. **I.** Proteins present in exosomes derived from control cells but absent in exosomes derived from SRGN-KD cells were subjected to gene ontology (GO) analysis. The negative logarithm of *P* values is shown for each GO term. The top 10 biological processes absent in SRGN-null exosomes are depicted.

We recently demonstrated that serglycin, which is commonly regarded as an intracellular PG, is constitutively secreted by myeloma cells, and can function extracellularly [18]. Since serglycin has long been considered to be present within intracellular granules/vesicles [19, 22-25], we sought to determine if serglycin is present in the intracellularly generated extracellular vesicles, exosomes, of myeloma patients. Exosomes were isolated from serum samples of relapsed myeloma patients using the ExoQuick PLUS exosome isolation kit. We and others have shown that the ExoQuick kit yields high-quality exosomes and can be used as an alternative to ultracentrifugation when limited amounts of biological samples are available [9, 30]. Electron microscopy (Figure 2A) and nanoparticle tracking analysis (Figure 2B) demonstrated that the particles isolated from serum were within the size range characteristic of exosomes (30-120 nm). By Western blotting we could detect serglycin in exosomes from most of the myeloma patients (Figure 2C). Serglycin was intact after chondroitinase ABC treatment (bacterial enzyme that degrades CS chains) of exosomes, indicating that serglycin is present inside the exosomes (Figure 2D). To determine if serglycin is present in exosomes that are released by myeloma cell lines, we purified exosomes from the conditioned medium of OCIMy5, CAG, and RPMI 8226 human myeloma cells using the gold standard ultracentrifugation method [9]. We have previously evaluated the efficacy of exosome purification using this method and will refer to pellets resulting from centrifugation at 100,000 × *g* as exosomes [12]. As expected, characterization of exosomes by nanoparticle tracking analysis revealed that they were relatively homogeneous with an average diameter of approximately 95 nm, consistent with the size range characteristic of exosomes (Figure 2E). Electron microscopy of negatively stained exosomes revealed a “cup shape” typical of exosomes (data not shown). As shown in Figure 2F, serglycin was found to be present in exosomes from all human myeloma cell lines tested, except in serglycin knocked down (SRGN-KD) CAG cells [18]. Serglycin ran as a broad smear on the western blot, ranging from 300 KDa to 140 KDa; this is typical of PGs and is due to variations in the number and length of CS GAG chains attached to the core protein of serglycin. Equal loading of exosomal protein was confirmed by probing for the exosomal marker protein clathrin.

### Serglycin modulates the protein cargo of myeloma-derived exosomes

Negatively charged GAG chains of serglycin mediate the storage of a variety of basically charged components within intracellular granules/vesicles [20]. To determine if serglycin regulates the secretion and protein composition of myeloma-derived exosomes, exosomes were isolated from conditioned medium from CAG control or SRGN-KD cells (these cells proliferate at the same rate *in vitro* [18]) by ultracentrifugation and analyzed by nanoparticle tracking analysis. The total number of exosome particles isolated did not differ significantly between SRGN-KD cells and control cells (Figure 2G). Further, the exosome particles from both cell types were consistently within the expected size range of exosomes (data not shown), suggesting that serglycin does not influence the biogenesis and size of exosomes.

To determine if serglycin regulates the protein composition of exosomes, exosomes were isolated from equivalent volumes of conditioned medium from control (referred to as SRGN-exosomes) and SRGN-KD (referred to as SRGN-null exosomes) myeloma cells, layered on the top of a 40% iodixanol cushion, and centrifuged. Material excluded by the iodixanol layer was subjected to mass spectrometry for proteomic analysis. Interestingly, the protein contents in SRGN exosomes and SRGN-null exosomes were very different. Significantly fewer proteins were present in SRGN-null exosomes than in SRGN-exosomes (Figure 2H). The Venn diagram in Figure 2H shows 223 proteins unique to the SRGN exosomes and 67 proteins unique to SRGN-null exosomes with 454 common proteins but not in SRGN-null exosomes. These findings corroborate the observations in serglycin-knockout animals, and confirm that serglycin is critical for maintaining the protein cargo in exosomes. However, further studies are required to understand whether the deficiency of protein components within SRGN-null exosomes is due to a defect in serglycin-mediated protein sorting to exosomes or a defect in serglycin mediated binding and retention of proteins within exosomes.

We next conducted a gene ontology (GO) enrichment analysis of the 223 proteins that were present in SRGN exosomes but not in SRGN-null exosomes using DAVID version 6.8 [31]. Interestingly, we found that, compared to SRGN exosomes, SRGN-null exosomes lacked proteins involved in translational initiation, cell adhesion, intracellular protein transport, and drug response pathways (Figure 2I). These findings demonstrated a critical role of serglycin in modulating the cargo of exosomes derived from CAG human myeloma cells.

Because we previously showed a critical role of serglycin in myeloma cell adhesion to bone marrow stromal cells and collagen I [18], we next focused on proteins involved in cell adhesion pathways. GO analysis identified 17 proteins involved in cell adhesion pathways that were present only in SRGN exosomes but not in SRGN-null exosomes (Supplementary Table 1). Among these missing proteins were the cell adhesion molecules CD44 and α4β1 integrin, which we have shown to play roles in myeloma cell adhesion [32, 33]. In addition we have also shown that myeloma-derived serglycin can bind to CD44 [18].

### Serglycin-null exosomes have less impact than serglycin containing exosomes in regulating the behavior of tumor and host cells

**Figure 3:**
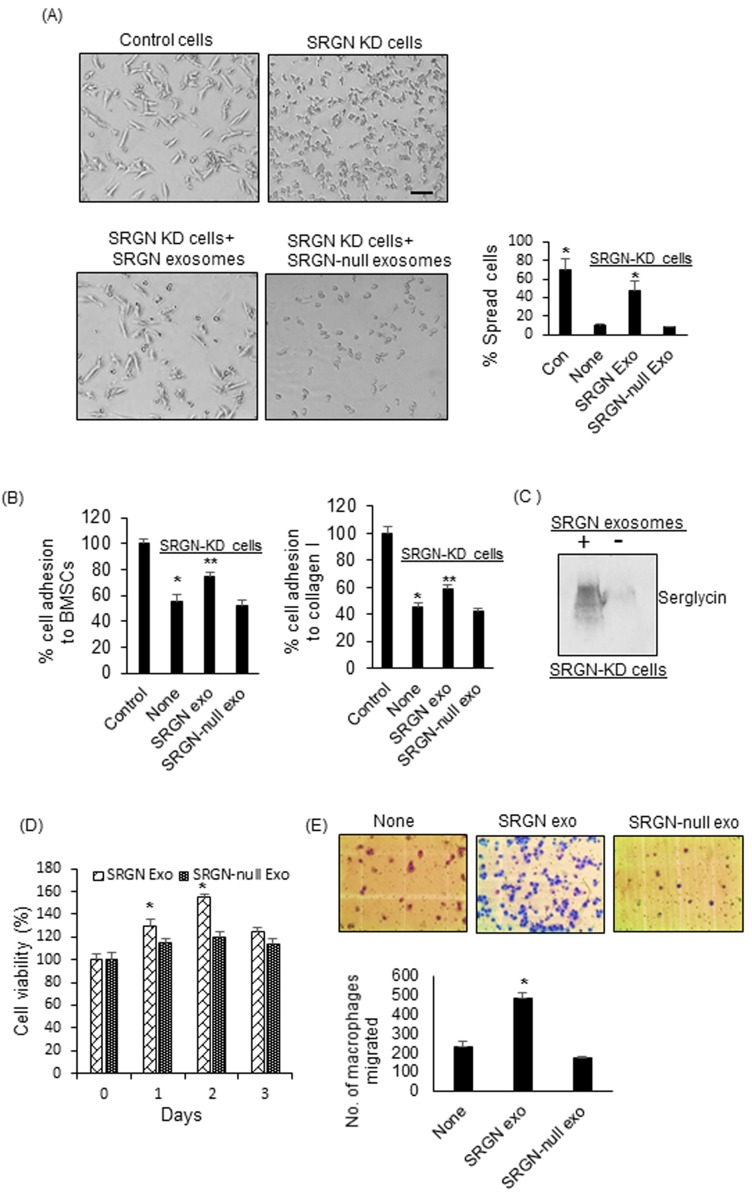
Serglycin null myeloma-derived exosomes have less impact on tumor and host cell behavior than serglycin containing exosomes **A.** Upper panel, control or SRGN-KD CAG cells were added to fibronectin-coated wells. After 12 h, the cells were washed, fixed, and photographed. Lower panel, 1x10^7^ SRGN or SRGN-null exosomes were added to SRGN-KD cells growing on fibronectin-coated wells. Following overnight incubation, the cells were washed, fixed, and photographed (bar = 50 µm). Results shown are representative panels from 2 different experiments using 2 different exosome preparations. Right panel represents the quantification of cell spreading on fibronectin. ^*^ *P* < 0.01 against SRGN-KD cells only (none). **B.** SRGN-KD CAG cells were exposed to SRGN exosomes (10^7^) or SRGN-null exosomes (10^7^) overnight and their adhesion to BMSCs and collagen I coated wells was analyzed. Values were normalized to control CAG cells, which were arbitrarily set at 100% (Mean ± SD of triplicates from three independent experiments). ^*^ *P* < 0.01 compared to control, ^**^ *P* < 0.05 compared to none. **C.** To analyze the exosome-mediated transfer of serglycin to recipient cells, SRGN-KD CAG cells were treated with or without SRGN exosomes. After overnight incubation, cells were lysed and analyzed for serglycin by western blotting. **D.** SRGN or SRGN-null exosomes (5x10^6^/ml) were added to wild-type CAG cells, and their growth was analyzed by MTT assay. ^*^P < 0.05 against SRGN-null exosomes. **E.** 5x10^6^ SRGN or SRGN-null exosomes were added to J774 murine macrophages, and their migration though an 8-micron-pore filter overnight was measured and photographed. Data are mean ± SD from 3 independent experiments. ^*^ *P* < 0.05 against no exosome addition (none).

Following the observation that serglycin can regulate the protein cargo of myeloma-derived exosomes, we studied the effect of SRGN exosomes and SRGN-null exosomes on tumor and host cell behavior. We previously demonstrated that myeloma-derived exosomes can impact the behavior of both tumor and host cells [9, 12]. Here, we hypothesized that SRGN-null exosomes have less impact than SRGN exosomes on recipient cell behavior. To test this, we first sought to determine the impact of these exosomes on myeloma cells. Although myeloma cells grow predominantly in suspension when in culture, we found that when cells were plated on fibronectin-coated plates, CAG control cells had a highly polarized morphology, whereas SRGN-KD cells failed to spread (Figure 3A). The polarized morphology of CAG control cells is typical of motile cells [32]. These findings suggested that serglycin promotes an invasive phenotype in myeloma cells. To test if SRGN exosomes, isolated from control CAG cells, could transfer this invasive phenotype to SRGN-KD cells, SRGN-KD cells were seeded on fibronectin-coated wells, to which were added, in equal amounts, purified SRGN exosomes or SRGN-null exosomes. We observed that SRGN exosomes induced a highly-polarized phenotype in SRGN-KD cells, whereas SRGN-null exosomes failed to elicit the same response (Figure 3A). Further, based on our pervious finding that serglycin can promote the adhesion of myeloma cells to bone marrow stromal cells (BMSCs) and collagen I, we also tested the impact of SRGN exosomes and SRGN-null exosomes on myeloma cell adhesion towards BMSCs and collagen I. As expected, SRGN-KD CAG myeloma cells showed significantly less adhesion to BMSCs and collagen I compared to control CAG cells (Figure 3B). Interestingly, prior exposure of SRGN-KD cells to SRGN exosomes enhanced the ability of these cells to bind BMSCs and collagen I (Figure 3B). However prior treatment with SRGN-null exosomes did not impact the adhesion of SRGN-KD cells to BMSCs and collagen I. We next assessed whether exosomes mediate the intracellular transfer of serglycin. SRGN-KD CAG cells were incubated with SRGN exosomes overnight, and the cells were lysed and analyzed for serglycin by western blotting. As shown in Figure 3C, higher levels of serglycin was detected in SRGN-KD cells exposed to SRGN exosomes than in exosomes untreated SRGN-KD cells. Given the important role of cell-extrinsic factors in cell growth, we next assessed whether SRGN and SRGN-null exosomes differentially impacted the proliferation of myeloma cells. Wild type CAG cells were incubated with SRGN exosomes or SRGN-null exosomes for different times, and cell viability was assessed by MTT assay. SRGN exosomes increased proliferation significantly after 1 to 2 days, compared to SRGN-null exosomes (Figure 3D).

Having confirmed the differential impact of SRGN and SRGN-null exosomes on tumor cells, we next assessed if these exosomes differentially impacted host cell behavior. Since myeloma-derived exosomes support the recruitment of macrophages, we investigated the effect of exosomes on the migratory properties of macrophages using a transwell migration assay. We found that, compared to no treatment, SRGN exosomes significantly increased the migration of macrophages through an 8-micron-pore membrane (Figure 3E). However, SRGN-null exosomes failed to stimulate the migration of macrophages (Figure 3E). Taken together, our findings suggested that depletion of serglycin from myeloma exosomes markedly reduces their impact on tumor and host cell behavior. Since we added equal number of SRGN-exosomes and SRGN-null exosomes in each of our functional assays, the difference we observed in their ability to alter tumor and host cell behavior can be solely traced back to their difference in cargo.

## DISCUSSION

Intercellular communication is a hallmark of multiple myeloma progression. Despite an emerging appreciation of the role of tumor-derived exosomes in mediating intercellular communication and tumor progression, no therapeutic strategies have been developed that target the impact of tumor exosomes. In the present study, we showed an important role of the CSPG serglycin in regulating the protein cargo and functions of myeloma-derived exosomes. We established for the first time that serglycin is present in exosomes derived from myeloma cell lines and the serum of myeloma patients. We also showed that knockdown of serglycin in myeloma cells decreased the number of exosomal proteins and reduced the impact of these exosomes on tumor and host cells. Our findings provide a novel mechanism by which an intracellular molecule regulates intercellular communication in myeloma.

We found that serglycin is required for maintaining the protein cargo of myeloma-derived exosomes, but not required for their biogenesis. Consistent with our findings, studies using serglycin knockout animals and their control littermates have shown that these animals have the same number of secretory granules in their mast cells, but their morphology are different [22, 34]. While control mice had granules filled with electron-dense materials, these type of granules were absent in serglycin knockout mice [22, 34]. Further, a more detailed transmission electron microscopy analysis of bone marrow cells revealed that, different from control animals, the exosomes in the bone marrow cell granules of serglycin-knockout mice were not filled with electron-dense material [22]. Our current findings, taken together with these previous studies, suggest that serglycin plays an important role in retaining the protein cargo within exosomes. Hence, serglycin in myeloma cells helps generate exosomes enriched with protein cargo that, when released into the tumor microenvironment, deliver their cargo to nearby or distant cells and thereby promote myeloma progression.

Previous studies have shown that serglycin binds and stores granular components within the secretory granules *via* serglycin’s negatively charged GAG chains [26, 27]. We and others have recently demonstrated that serglycin expressed by myeloma cells is decorated only by CS chains and that these CS chains are almost completely composed of 4-*O*-sulfated disaccharides [18, 21]. Interestingly, gene array analysis found that the expression of *CHST11* (also known as *C4ST1*), a sulfotransferase gene responsible for the 4-*O*-sulfation of CS chains, is highly upregulated in myeloma patients compared to healthy donors [35]. Since the anionic charge density of serglycin regulates the exosomal protein cargo, it is possible that altering the levels or activity of *CHST11* could shut down sulfation of CS chains and thereby decrease the protein cargo and functions of exosomes. Therefore, further studies are required to understand whether modulating the anionic charge density on the CS chains of serglycin by silencing *CHST11* expression myeloma cells may be an alternative approach to target myeloma exosomes. We would also like to stress that, though anionic GAGs of serglycin may be responsible for the storage of proteins within exosomes, we are still not sure whether this accounts for the absence of the 223 proteins in SRGN-null exosomes. We therefore speculate that serglycin may also regulate the exosomal protein cargo by additional mechanisms, such as by regulating the protein sorting pathway or by binding and retaining exosomal proteins through their core protein. Studies have shown that the core protein of serglycin from myeloma cells can bind to proteases like MMP-9 (matrix metalloproteinase-9) and MMP-13 [36-38].

GO analysis showed that the biological pathways associated with the proteins absent from SRGN-KD exosomes were mainly in categories such as translation, cell adhesion, RNA processing, and intracellular protein transport. These pathways were previously reported to be enriched in exosomes [39]. Our findings, therefore, indicate that elimination of serglycin from myeloma cells eliminates specific exosomal proteins that are capable of activating critical biological processes in target cells. For example, our results showed for the first time that SRGN exosomes but not SRGN-null exosomes can promote the proliferation of myeloma cells, can induce an invasive phenotype in myeloma cells, and can increase the migration of macrophages (Figure 3). These findings suggest that the serglycin proteoglycan is responsible for retaining specific cargo within myeloma-derived exosomes and that, once these exosomes interact with a target cell, serglycin and its binding partners are delivered to these cells and thereby reprogram the target cells to support cancer progression.

## MATERIALS AND METHODS

### Cell lines and antibodies

RPMI 8226, OCIMy5, and CAG human myeloma cells were cultured as described previously [18]. SRGN-KD and control myeloma cells were engineered as described previously [18]. Serglycin and clathrin antibodies were obtained from Sigma and Abcam, respectively. CD63 antibody from Santa Cruz.

### Exosome isolation

Exosomes from cell culture supernatants (by ultracentrifugation) and serum of myeloma patients were isolated and characterized using electron microscopy and nanoparticle tracking analysis using a ZetaView analyzer, as described by us previously [9]. Exosomes from 250 µl of serum of relapsed myeloma patients were isolated using the ExoQuick PLUS exosome isolation kit (System Bioscience), which uses microsphere beads for additional purification of exosomes. For some experiments, a subset of patient derived exosomes (1x10^7^) where incubated with protease-free bacterial chondroitinase ABC (2U/ml) (Chase ABC, Sigma) for 2h at 37°C before use in western blotting for serglycin. For mass spectrometry analyses, equal numbers of exosomes obtained by ultracentrifugation were layered on the top of a 40% iodixanol cushion (Sigma) and centrifuged for 16 h at 28,000 rpm. The remaining exosome fraction excluded by the cushion was pelleted by ultracentrifugation at 100,000 × g for 70 min and used for analysis, as described [9].

### Bioinformatics analysis

Protein identifications by LC-MS/MS analysis were accepted if they could be established at > 90.0% probability and contained at least 2 identified peptides. Peptides with zero counts were considered absent in exosomes. GO enrichment analysis was performed using DAVID (Database for Annotation, Visualization and Integrated Discovery) version 6.8 [31] to understand the biological role of proteins that were present in control exosomes but absent from SRGN-KD exosomes. A functional annotation chart was derived with default parameters (gene count of 2 and EASE of 0.1). GO biological processes statistically enriched in a given gene set (*P* < 0.05) were identified.

### Exosome functional assays

The role of exosomes in myeloma cell spreading and adhesion to BMSCs and collagen I were studied as described previously [12, 18]. Migration of macrophages was determined using 8-micron-pore filters (BD Bioscience) as described previously [9]. For myeloma cell proliferation assays, exosomes (5x10^6^ particles/ml) were added to CAG cells, and proliferation was measured using the MTT reagent.

## SUPPLEMENTARY MATERIALS TABLE



## Abbreviations

SRGN: serglycin
PG: proteoglycan
CS: chondroitin sulfate
C4ST1: chondroitin 4-*O*-sulfotransferase-1
GO: Gene Ontology
GAG: glycosaminoglycan
BMSCs: bone marrow stromal cells
