# Supplementary material for: Chondroitin sulfate proteoglycan serglycin influences protein cargo loading and functions of tumor-derived exosomes
Source: Oncotarget. 2017 Aug 27;8(43):73723–32. doi: 10.18632/oncotarget.20564 (PMC5650294; doi:10.18632/oncotarget.20564)
Supplement: Supplementary file 1 [file oncotarget-08-73723-s001.pdf]

Chondroitin sulfate proteoglycan serglycin influences protein cargo loading and functions of tumor-derived exosomes

SUPPLEMENTARY MATERIALS

Table 1: List of proteins involved in cell adhesion pathway that are absent in exosomes from serglycin knockdown myeloma cells.

CD44
Integrin alpha-4
Integrin beta-1
Poly(U)-binding-splicing factor PUF60
Septin-9
Protein S100-A11
ATP-dependent RNA helicase DDX3X
14-3-3 protein zeta/delta
Protein DJ-1
General vesicular transport factor p115
60S ribosomal protein L29
EH domain-containing protein 4
Eukaryotic translation initiation factor 2A
Plectin
Septin-2
EH domain-containing protein 1
Plasminogen activator inhibitor 1 RNA-binding protein

Gene ontology enrichment analysis was performed using DAVID (Database for Annotation, Visualization and Integrated Discovery) v6.8 to understand the biological role of proteins which were found to be exclusively absent in SRGN null exosomes compared to SRGN exosomes. Functional annotation chart was derived with default parameters [gene count of 2 and EASE of 0.1] and proteins involved in cell adhesion pathway that are absent in SRGN null exosomes compared to SRGN exosomes were identified (P-value <0.05).
